# The eicosanoids leukotriene D_4_ and prostaglandin E_2_ promote the tumorigenicity of colon cancer-initiating cells in a xenograft mouse model

**DOI:** 10.1186/s12885-016-2466-z

**Published:** 2016-07-07

**Authors:** Kishan Bellamkonda, Naveen Kumar Chandrashekar, Janina Osman, Benson Chellakkan Selvanesan, Sayeh Savari, Anita Sjölander

**Affiliations:** Division of Cell and Experimental Pathology, Department of Translational Medicine, Lund University, Clinical Research Center, Skåne University Hospital, SE-205 02 Malmö, Sweden

**Keywords:** Colon cancer, PGE_2_, LTD_4_, ALDH, Inflammation, Cancer-initiating cells

## Abstract

**Background:**

Colorectal cancer is one of the most common types of cancers worldwide. Recent studies have identified cancer-initiating cells (CICs) as a subgroup of replication-competent cells in the development of colorectal cancer. Although it is understood that an inflammation-rich tumor microenvironment presumably supports CIC functions, the contributory factors are not very well defined. The present study advances our understanding of the role of the eicosanoids leukotriene D_4_ (LTD_4_) and prostaglandin E_2_ (PGE_2_) in the tumorigenic ability of CICs and investigates the consequential changes occurring in the tumor environment that might support tumor growth.

**Methods:**

In this study we used human HCT-116 colon cancer ALDH^+^ cells in a nude mouse xenograft model. Protein expression and immune cell was determined in tumor-dispersed cells by flow cytometry and in tumor sections by immunohistochemistry. mRNA expressions were quantified using RT-q-PCR and plasma cytokine levels by Multiplex ELISA.

**Results:**

We observed that LTD_4_ and PGE_2_ treatment augmented CIC-induced tumor growth. LTD_4_-and PGE_2_-treated xenograft tumors revealed a robust increase in ALDH and Dclk1 protein expression, coupled with activated β-catenin signaling and COX-2 up-regulation. Furthermore, LTD_4_ or PGE_2_ accentuated the accumulation of CD45 expressing cells within xenograft tumors. Further analysis revealed that these infiltrating immune cells consisted of neutrophils (LY6G) and M2 type macrophages (CD206^+^). In addition, LTD_4_ and PGE_2_ treatment significantly elevated the plasma levels of cysteinyl leukotrienes and PGE_2_, as well as levels of IL-1β, IL-2, IL-6, TNF-α and CXCL1/KC/GRO. In addition, increased mRNA expression of IL-1β, IL-6 and IL-10 were detected in tumors from mice that had been treated with LTD_4_ or PGE_2_.

**Conclusion:**

Our data suggest that both LTD_4_ and PGE_2_ promote CICs in initiating tumor growth by allowing modifications in the tumor environment. Our data indicate that new therapeutic strategies targeting eicosanoids, specifically LTD_4_ and PGE_2_, could be tested for better therapeutic management of colon cancer.

**Electronic supplementary material:**

The online version of this article (doi:10.1186/s12885-016-2466-z) contains supplementary material, which is available to authorized users.

## Background

Colorectal cancer (CRC) is a major healthcare burden and the fourth most common cause of cancer-related deaths in the Western world [1, 2]. The etiological factors and pathogenic mechanisms underlying the development of CRC are complex and heterogeneous [3]. Many studies have demonstrated the pre-existing inflammatory milieu as the main cause for CRC progression [4]. The best characterized example for the role of inflammation in cancer is manifested by inflammatory bowel disease cases where long-standing inflammation imposes a high risk of CRC development [5, 6]. Moreover, non-steroidal anti-inflammatory drugs (NSAIDs) reduce the long-term risk of cancer death, highlighting the importance of inflammation in cancer progression [7]. It is believed that chronic inflammation facilitates tumor progression by establishing a milieu that promotes the growth of cancerous cells. Inflammatory cells recruited to inflammatory foci can release various pro-inflammatory mediators, including eicosanoids and cytokines, which can change the microenvironment to an abnormal milieu. An increase in immune cells can alter the microenvironment so that it becomes pro-inflammatory and acquires the capability to change the phenotype of epithelial cells to promote tumor growth and metastasis [8].

Several studies have demonstrated that eicosanoids, such as prostaglandins and leukotrienes, are important inflammatory mediators in the crosstalk between epithelial cells and the surrounding stromal cells in the tumor microenvironment [9, 10]. The importance of COX-2-derived PGE_2_ in tumor progression is well proven in mouse models of CRC [11–13]. PGE_2_ treatment has been shown to increase intestinal polyps in both Apc^Min/+^ mice and AOM-induced mice [13]. Interestingly, the LTD_4_ receptor CysLT_1_R is highly expressed in human colon cancer and correlates negatively with patient survival [14, 15]. Moreover, LTD_4_ was found to induce proliferation and survival [16]. By contrast, reduced expression of CysLT_2_R is associated with a poor prognosis in patients with CRC, and CysLT_2_R signaling promotes apoptosis and differentiation [14, 17]. Taken together, these studies show the key role of eicosanoids in CRC development.

Over the last decade, the emergence of new therapeutic targets has improved cancer therapy and has prolonged the lifespan of these patients. However, initial therapy and recovery are often complicated by the development of relapsed tumors. Cancer-initiating cells (CICs) are believed to be a small group of tumor cells that can form tumors [18]. CICs have been identified in many different tumors such as those in the hematopoietic system, breast, brain, head, neck, and colon [19–22]. One important characteristic of CICs is their self-renewal capacity. Therefore, CICs are the most probable cause of tumor chemoresistance and recurrence, and may be accountable for the current failure of standard therapies [23, 24].

CICs can be identified by inducing stemness-selective conditions, or isolated based on the putative expression of stem cell markers. Multiple cell surface markers like CD133, CD44, CD24, CD29, CD166 and Lgr5 have been used by different investigators to isolate CIC subpopulations from colon carcinomas [20, 25–27]. Besides cell surface markers, the activity of certain pathways or enzymes is also used to identify stemness in cells. The activity of aldehyde dehydrogenase 1 (ALDH1), a detoxifying enzyme that oxidizes intracellular aldehydes, has also been used to identify normal colon stem cells [28]. In a recent study, we showed that ALDH^+^ cells, compared with other cell surface markers, such as CD133 and CD44, could initiate more colonies than ALDH^−^ cells, whereas this distinction was not apparent in positive and negative cells of CD133 and CD44 [29]. This finding indicates that ALDH activity can be considered a reliable CIC marker.

Furthermore, in a recent study, we have shown that inflammatory lipid mediators can actually enhance the characteristic properties of CICs under certain conditions in vitro [29].

The aim of the current study was to elucidate whether enrichment of the tumor microenvironment by inflammatory lipid mediators such as LTD_4_ and PGE_2_ could promote the tumorigenic properties of CICs in vivo and to determine the changes occurring in the tumor environment that could modify CIC functions.

## Methods

### Reagents and antibodies

LTD_4_, PGE_2_, rabbit anti-human COX-2 and 5-LOX polyclonal antibodies were purchased from Cayman Chemical (Ann Arbor, MI, USA). The ALDEFLOUR (ALDH) kit was purchased from Stem Cell Technologies (Grenoble, France). Anti-human CD326 (EpCAM) MicroBeads was obtained from Miltenyi Biotec (Gladbach, Germany). Anti-mouse CD45-FITC-conjugated antibody was purchased from Santa Cruz Biotechnology (Santa Cruz, CA). Anti-mouse LY6G-PE, CD4-PE, and F4/80-PE conjugated antibodies were purchased from BD Biosciences (Franklin Lakes, NJ, USA). Anti-mouse CD206-Alexaflour 647 conjugated antibody was obtained from AbD Serotech (Dusseldorf, Germany). Rabbit anti human β-catenin antibody, mouse anti-human ALDH and matrigel basement membrane matrix were obtained from BD Biosciences (Franklin Lakes, NJ, USA). The rabbit monoclonal anti-human Ki67 antibody was obtained from Thermo Fisher Scientific (Waltham, MA). Rabbit anti-human Dclk1 antibody and Rat anti-mouse F4/80 antibody were obtained from Millipore (Temecula, CA, USA) and AbD serotec (Raleigh, NC, USA), respectively. All other chemicals were of analytical grade and were obtained from Chemicon International (Temecula, CA) or Sigma Chemical Co. (St. Louis, MO) unless otherwise stated.

### Cell culture

HCT-116 cells (ATCC# CCL-247), derived from human colon carcinoma, were obtained from the American Type Culture Collection (ATCC, Manassas, VA). The cells were maintained in monolayer culture in McCoy’s 5A modified medium (Gibco BRL, Grand Island, NY) supplemented with 10 % fetal bovine serum (FBS), 55 μg/ml streptomycin and 55 IU/ml penicillin. The cells were grown until 5 days to 70–80 % confluence at 37 °C in a humidified atmosphere of 5 % CO_2_. The cell line tested negative for mycoplasma (MycoTect® kit, Gibco BRL) at regular intervals.

### Flow cytometry

FACS was used to sort the ALDH^+^ cells from the parental HCT-116 cell line for xenograft implantation and to quantify the presence of CD45^+^ and ALDH^+^ subpopulations in dissociated tumor cells. For FACS sorting of ALDH^+^ cells, HCT-116 cells were harvested using 0.25 % trypsin and 0.02 % EDTA. After resuspension of the cells in serum-free culture media, the cells were washed with 0.5 % BSA/PBS and stained with Aldefluor reagent (Stem Co Biomedical, Durham, NC, USA). The Aldefluor substrate was added to 1 × 10^6^ cells/ml suspended in Aldefluor assay buffer and incubated at 37 °C for 40 min. At the same time, cells treated with the specific ALDH inhibitor diethylaminobenzaldehyde (DEAB) were stained to serve as the negative control. Cells with bright fluorescent ALDH signals were detected using a FACSCalibur or FACSAria flow cytometer (BD Biosciences). The ALDEFLUOR kit was used to sort ALDH^+^ cells with high ALDH enzymatic activity, as described previously [29, 30].

For analysis of CD45^+^ cells, single-cell suspensions from digested tumors were washed, resuspended in PBS, counted and divided into 1 × 10^6^ cell aliquots for flow cytometry. Cells were washed again with 0.5 % BSA/PBS, resuspended in 100 μl of PBS with 1 % mouse serum, and incubated at 4 °C for 45 min for Fc-receptor blocking. Thereafter, each sample was exposed to 5 μl of anti-mouse CD45-FITC antibody for 45 min at 4 °C in the dark. Cells were washed with 0.5 % BSA/PBS, resuspended in 100 μl of PBS with 0.1 % μg/μl of 7-AAD (BD Pharmingen), and incubated at 4 °C for 10 min in the dark. Finally, 400 μl of PBS was added to each sample and read on a flow cytometer. The analysis was performed using the Summit v4.6.

### Xenograft tumors

The 5- to 6-week-old female nude mice (BalbC nu/nu) used in this study were purchased from Taconic Europe A/S (Ry, Denmark). The Regional Ethical Committee for Animal Research at Lund University, Sweden (M401-12) approved the animal experiments. To induce subcutaneous human colon cancer xenografts, FACS-sorted 1 × 10^4^ ALDH^+^ HCT-116 cells were suspended in a 1:1 mixture of PBS:Matrigel (BD Biosciences), and 100 μl of the mixture was injected subcutaneously into each of both flanks of the mice. Tumor development was detected by palpation. The time taken for a palpable tumor to develop was recorded (10–14 days), and the tumor size was measured every three days using a digital vernier caliper. Once palpable tumors were established, the mice were randomly divided into three groups, and then were treated with vehicle, LTD_4_ or PGE_2_. The mice received daily subcutaneous injections of either ethanol (5 %) as vehicle, or 24.8 μg/kg/day of LTD_4_ or 17.6 μg/kg/day of PGE_2_. Tumor growth was monitored, and the tumor volume was estimated every third day. All mice were sacrificed after 48 days. The tumors were removed, measured, weighed, and photographed. Tumor tissues were fixed in 10 % buffered formalin, embedded in paraffin for immunohistochemistry analysis and/or processed further for tissue dissociation immediately for FACS analysis. Tumor volumes were estimated according to the formula (length × width^2^)/2.

### Dissociation of xenograft tumors

After sacrificing the mice, the excised tumors were washed with PBS and minced using sterile scalpels. The minced tumor pieces were resuspended in RPMI medium supplemented with 10 % FBS and 2 mg/ml Collagenase P (Roche diagnostics, Basel, Switzerland). The tumor pieces were further dissociated using the gentleMACS™ Dissociator for 30 s and incubated at 37 °C for 2 h with rotation. Afterwards, the cell suspension was filtered through a 70-μm mesh and washed once with PBS and counted. The single cells were separated into mouse cells and epithelial tumor cells using CD326 (EpCAM) microbeads. The mouse cells and epithelial tumor cells were immediately stained for CD45, CD4, LY6G, F4/80, CD206 and ALDH, respectively, for FACS analysis.

### Immunohistochemistry

Paraffin-embedded sections obtained from xenograft tumors were sectioned (4 μm) for immunohistochemical staining. All procedures were performed using a Dako automatic slide stainer according to the manufacturer’s instructions. Tumor sections were treated with 1–3 % hydrogen peroxide, blocked, and incubated with anti-ALDH, anti-COX-2 or anti-F4/80 (1:100 dilution each), anti-Dclk1 or anti-5-LOX (1:200 dilution each), or anti-β-catenin (1:300). Sections were incubated with biotinylated secondary antibody, followed by ABC reagent (Vector Laboratories Inc., Burlingame, CA). Signals were detected using DAB solution (Vector Laboratories). Tissues were counterstained with hematoxylin. The slides were scanned using the Aperio ScanScope CS system (Aperio Technologies Inc, Vista, CA, USA), and images were evaluated in a blinded fashion by two independent observers.

The immunoreactivity of β-catenin, COX-2, 5-LOX and F4/80 proteins in the tumor cells was determined based on the following procedure. Briefly, staining intensity was scored as 0 (negative), 1 (very weak), 2 (weak), 4 (medium) or 6 (strong). The extent of staining was scored as 0 (0 %), 0.5 (1–5 %), 1 (6–10 %), 2 (11–20 %), 3 (21–30 %), 4 (31–40 %), 5 (41–50 %), 6 (51–60 %), 7 (61–70 %), 8 (71–80 %), 9 (81–90 %) and 10 (91–100 %) according to the percentage of the positive staining area in relation to the whole carcinoma area. Next, the sum of the intensity score and extent score was regarded as the final staining scores for COX-2, 5-LOX, F4/80 and β-catenin proteins.

### CysLTs, PGE_2_ and cytokine ELISA analysis

Blood was collected by cardiac puncture at the time of animal sacrifice, and a nonselective COX inhibitor, indomethacin, was immediately added to the blood samples together with the anticoagulant sodium citrate. The plasma was separated by spinning the samples at 5000 × g for 4 min. CysLTs and PGE_2_ plasma samples were measured using a competitive enzyme immunoassay obtained from Enzo Life Sciences (Solna, Sweden). All measurements were performed according to the manufacturer’s instructions.

Plasma cytokines were analyzed using a multiplex sandwich immunoassay format and the electro-chemiluminescence MSD ultrasensitive proinflammatory multiplex kit (Meso-Scale Discovery, Gaithersburg, MD). The MSD multispot array was run according to the manufacturer’s protocol. Briefly, 96-well plates pre-coated with capture antibodies for TNFα, IL-1β, IL-2, IL-4, IL-6, IL-10 and CXCL1/KC/GRO, INFγ were incubated with plasma samples for 2 h. Subsequently, detection antibodies were added, and the plate was incubated for another 2 h. After washing, the plate was read using an MS2400 imager (MSD).

### Real-Time quantitative PCR

qPCR reactions employing TaqMan gene expression assays were used to measure tumor tissue expression of CysLT1R (Hs00272624_s1), PTGER2 (Hs00168754_m1), PTGRR4 (Hs00168761_m1), Arginase 1 (Mm00475988_m1), IL-1β (Mm00434228_m1; Hs00174097_m1), IL-6 (Mm00446190_m1; Hs00985639_m1) and IL-10 (Mm01288386_m1) genes (Applied Biosystems, Cambridge, United Kingdom) as described earlier [29].

### Statistical analysis

Statistical analysis was performed using GraphPad Prism 5 software. Results are expressed as the mean ± SEM. All comparisons between the mean values were performed using either one-way analysis of variance (ANOVA) with Newman-Keuls post-hoc test, two-way ANOVA, or Student’s unpaired *t* test wherever applicable. *P* values less than 0.05 were considered to indicate statistical significance.

## Results

### Both LTD_4_ and PGE_2_ affect the tumorigenic potential of ALDH^+^ cells

**Fig. 1:**
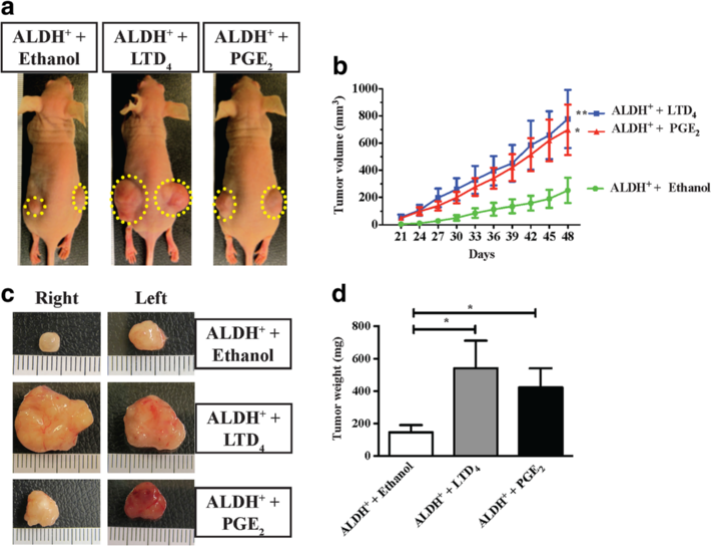
Effect of LTD_4_ and PGE_2_ on xenograft tumor growth initiated by ALDH^+^ HCT-116 cells. Mice were injected subcutaneously with 1 × 10^4^ ALDH^+^ HCT-116 cells into two flanks and received subcutaneous injections of vehicle (5 % ethanol in PBS), LTD_4_ (24.8 μg/kg/day) or PGE_2_ (17.6 μg/kg/day) from the third week onwards daily. **a** Images of xenograft mice with representative tumor sizes upon daily administration of either ethanol, LTD_4_ or PGE_2_ at day 48. **b** Graph showing tumor volume for the mice treated with vehicle (ethanol), LTD_4_ or PGE_2_. **c** Representative tumor images from treated groups at the experimental end-point, day 48. **d** Tumor weights of the LTD_4_- and PGE_2_-treated groups compared with the vehicle group at the end-point, day 48. The data shown are the means ± SEM, n = 6 mice in each group. *P < 0.05, **P < 0.01

In a recent in vitro study, we showed that an ALDH^+^ subpopulation of colon cancer cells is enriched with properties of cancer-initiating cells, and is increased two-fold in the presence of inflammatory lipid mediators such as LTD_4_ or PGE_2_ [29]. In this study we also investigated and observed that treatment with these two lipid mediators for 39 weeks increased tumor growth in a xenograph model [29]. To further study the effect of the microenvironment on the in vivo tumorigenicity of ALDH^+^ cells in the presence of LTD_4_ or PGE_2_, we injected HCT-116 ALDH^+^ cells in both flanks of nude mice. The mice received daily treatment of LTD_4_ or PGE_2_ to create an inflammation-enriched tumor microenvironment for a period of 48–49 days. Tumor growth was monitored every three days until the experimental endpoint after 48–49 days. As shown in Fig. 1, panel b, both LTD_4_ and PGE_2_ treatments significantly enlarged the tumor volume compared with the vehicle (ethanol)-treated ALDH^+^ group, results similar to those previously reported [29]. In addition, the tumor weight was significantly increased in both LTD_4_- and PGE_2_-treated mice compared with the vehicle-treated ALDH^+^ group (Fig. 1, panel d). Taken together, our data on the tumor growth, their size and weight indicated that both LTD_4_ and PGE_2_ could modulate the tumor environment of ALDH^+^ cells in favor of augmented tumor growth.

### Both LTD_4_ and PGE_2_ stimulation increase the percentage of ALDH^+^ cells and Dclk1, β-catenin and COX-2 protein expression

**Fig. 2:**
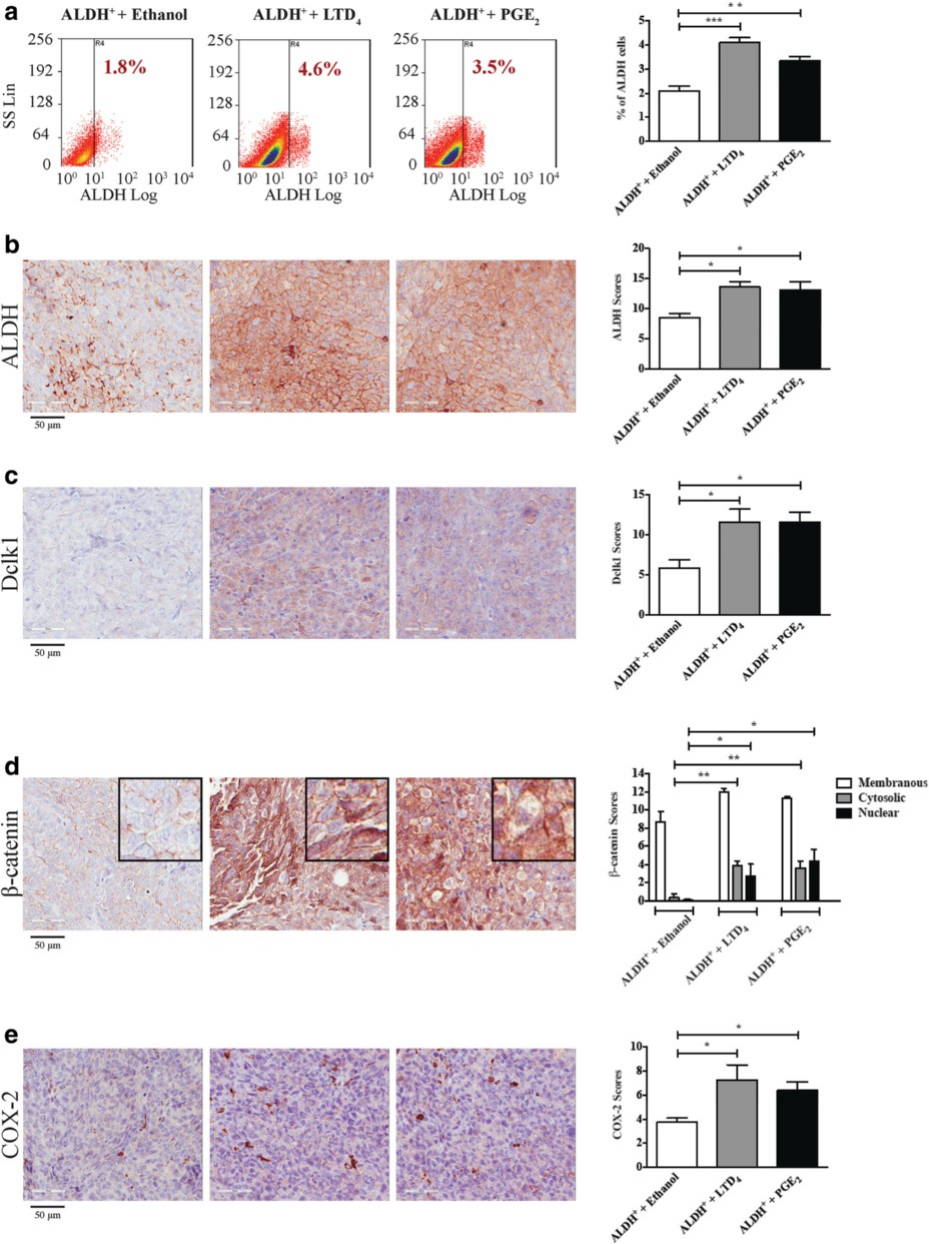
FACS analysis and immunohistochemistry of xenograft ALDH^+^ HCT-116 cell tumors treated with LTD_4_ or PGE_2_. **a** Representative dot plots and corresponding graphs of FACS analysis of the percentage of ALDH^+^ cells in dissociated tumors from vehicle (ethanol), LTD_4_ or PGE_2_ treated groups respectively. **b**–**e** Immunohistochemical analysis of ALDH, Dclk1, β-catenin, and COX-2 protein expression levels. The tumors from vehicle- (ethanol), LTD_4_- and PGE_2_-treated mice were processed for immunohistochemical analysis. Representative images (40×) and corresponding bar graphs show staining scores of (**b**) ALDH, (**c**) Dclk1, (**d**) β-catenin, and (**e**) COX-2 proteins in tumors. The final scores represent the sum of the staining intensity and staining percentage within tumor areas. The data are expressed as means ± SEM, n = 6 mice in each group. *P < 0.05, **P < 0.01 and ***P < 0.001

Furthermore, to ascertain how LTD_4_ or PGE_2_ facilitates tumor growth, we examined the percentage occurrence of different cell types, particularly CICs, within HCT-116 ALDH^+^ cell tumor sections. Interestingly, mice treated with LTD_4_ or PGE_2_ showed significantly higher percentage of ALDH^+^ cells in their tumors compared with the vehicle-treated ALDH^+^ group by FACS (Fig. 2, panel a). Similarly, increased protein expression of ALDH was seen in IHC sections of both LTD_4_ and PGE_2_ treated tumors of mice compared to control group (Fig. 2, panel b). Moreover we also found increased expression levels of the Dclk1 protein, an intestine cancer stem cell marker [31], within tumor sections from both LTD_4_- and PGE_2_- treated mice (Fig. 2, panel c). Further, to identify the factors influencing the LTD_4_- or PGE_2_-elicited tumor growth, we examined the protein level of β-catenin, COX-2, and 5-LOX. As summarized in Fig. 2 panel d and e, in vehicle-treated tumor sections, β-catenin was localized predominantly in the plasma membrane. However, both LTD_4_- and PGE_2_-treated tumors demonstrated significantly higher cytoplasmic β-catenin levels and increased nuclear localization than the control vehicle group. Furthermore, the COX-2 levels were significantly augmented in mice treated with LTD_4_ or PGE_2_ (Fig. 2, panel e). 5-LOX protein expression was not found to be significantly changed in mice treated with either LTD_4_ or PGE_2_ compared with the control vehicle group (Additional file 1: Fig. S1).

### Effect of LTD_4_ or PGE_2_ treatment on immune cells

**Fig. 3:**
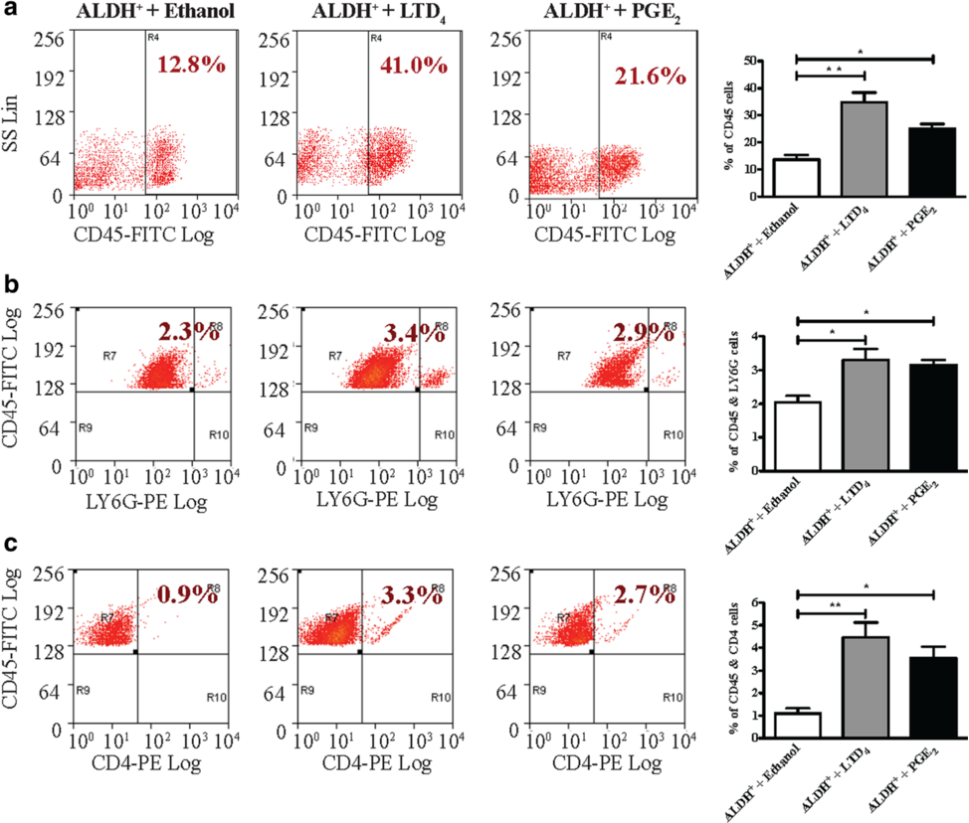
FACS analysis of xenograft ALDH^+^ HCT-116 cell tumors treated with LTD_4_ or PGE_2_. **a**–**c** Representative dot plots and corresponding graphs of FACS analysis of the percentage of CD45^+^ cells in dissociated tumors from vehicle (ethanol), LTD_4_ or PGE_2_ treated groups respectively. **a** CD45^+^, (**b**) LY6G, and (**c**) CD4^+^ cells in dissociated tumors from vehicle (ethanol), LTD_4_ or PGE_2_ treated groups respectively. The data are expressed as means ± SEM, n = 6 mice in each group. *P < 0.05, **P < 0.01 and ***P < 0.001

**Fig. 4:**
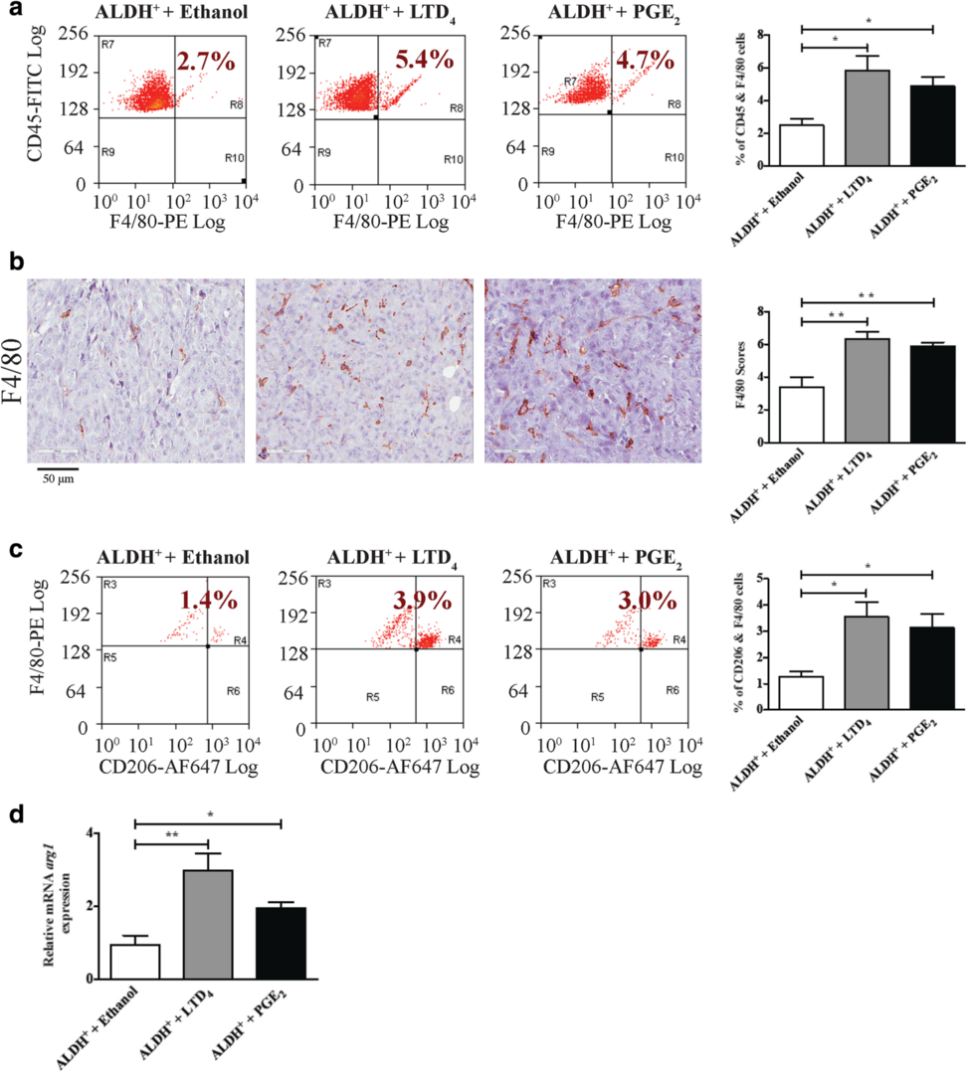
FACS and immunohistochemistry analysis of LTD_4_- or PGE_2_-treated xenograft mice tumors. **a** Representative dot plots and corresponding graphs of FACS analysis of the percentage of CD45^+^ and F4/80^+^ double positive cells in dissociated tumors from vehicle (ethanol), LTD_4_ or PGE_2_ treated groups respectively. **b** Representative immunohistochemistry images (40×) of F4/80 and their corresponding bar graphs show overall F4/80 scores within tumors. The final scores represent the sum of the staining intensity and staining percentage within the tumor area. **c** Representative dot plots and corresponding graphs of FACS analysis of the percentage of F4/80^+^ and CD206^+^ double positive cells in dissociated tumors from vehicle (ethanol), LTD_4_ or PGE_2_ treated groups respectively. **d** Relative mRNA expression of arginase 1 in tumors of mice treated with LTD_4_ or PGE_2_. The analyzed data are expressed as means ± SEM, n = 6 mice in each group. *P < 0.05 and **P < 0.01

Moreover, tumors from mice treated with LTD_4_ or PGE_2_ revealed by FACS analysis significantly increased percentage of CD45^+^ mouse cells compared with tumors from vehicle-treated mice (Fig. 3, panel a). Double staining of CD45^+^ cells for other immune cells markers revealed increased percentage of LY6G^+^ cells, neutrophils (Fig. 3, panel b), and CD4^+^ cells (Fig. 3, panel c). Further FACS analysis with double staining of CD45^+^ cells and macrophage marker F4/80 revealed increased percentage of macrophages in both LTD_4_- and PGE_2_-treated mice groups (Fig. 4, panel a). Because we observed by FACS analysis that LTD_4_- and PGE_2_-treated mice had an increased percentage of F4/80^+^ cells, we further analyzed the tumor sections by IHC examining the level of F4/80. We observed a significant increase in F4/80 protein expression in both the LTD_4_- and PGE_2_-treated groups compared with the control vehicle group (Fig. 4, panel b). Additionally, FACS gating F4/80 cells for CD206^+^ (a M2 macrophage marker) revealed increased percentage of CD206^+^ cells in tumors from mice treated with LTD_4_ or PGE_2_ compared with tumors from control mice (Fig. 4, panel c). We also detected increased arginase 1 (known to be highly expressed in M2 macrophages) expression in tumors from mice treated with LTD_4_ and PGE_2_ (Fig. 4, panel d), which corroborates our FACS data. These results provide support for a positive correlation between immune cells populating the tumor and CICs.

### Both LTD_4_ and PGE_2_ treatment increase IL-1 and IL-6 mRNA and cytokine secretion

**Fig. 5:**
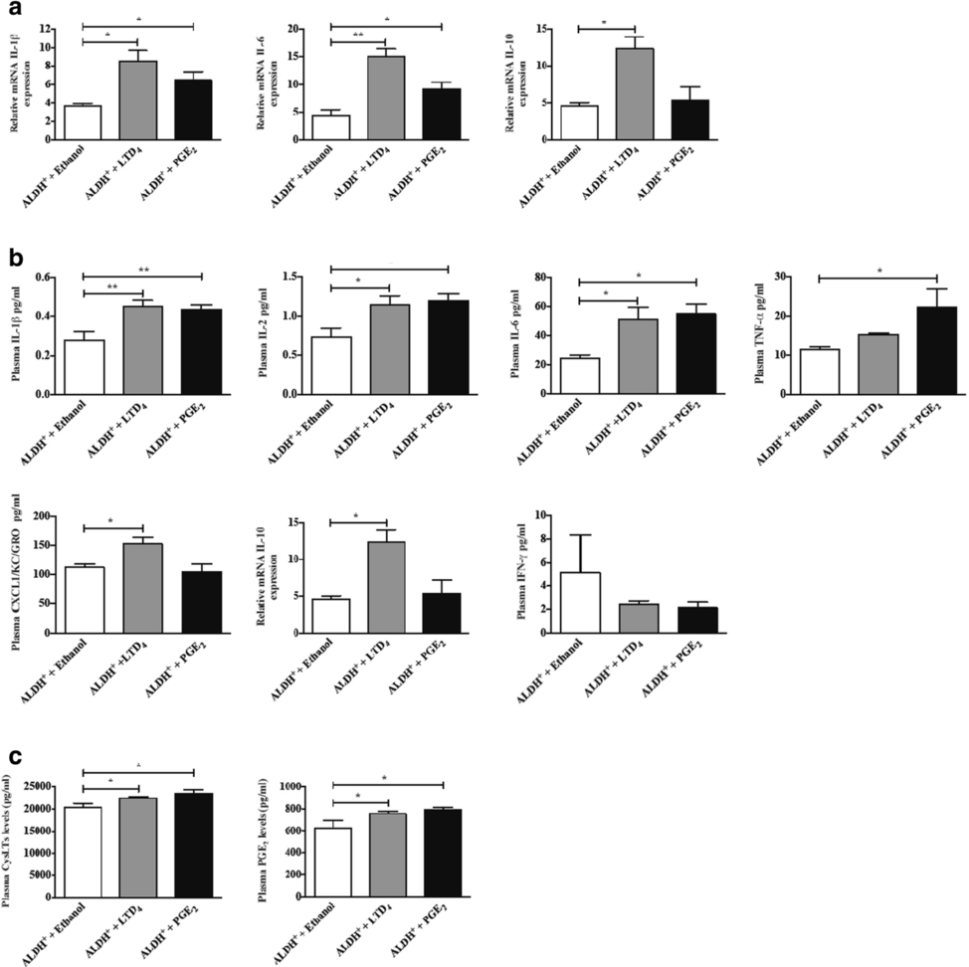
Effect of LTD_4_ or PGE_2_ on the levels of different cytokines and eicosanoids in xenograft ALDH^+^ HCT-116 cell tumor mice. **a** mRNA expression of cytokines (IL-1β, IL-6 and IL-10) in tumors of mice treated with vehicle, LTD_4_ or PGE_2_. **b** Plasma levels of cytokines (IL-1β, IL-2, IL-6, TNF-α, CXCL1, IL-10 and IFN-γ) from vehicle- (ethanol), LTD_4_- or PGE_2_-treated xenograft mice. **c** Plasma levels of CysLTs and PGE_2_ from vehicle, LTD_4_- or PGE_2_-treated xenograft mice. The results are expressed as means ± SEM, n = 6 mice in each groups. *P < 0.05 and **P < 0.01

We next investigated the tumor mRNA expression of some key cytokines, we observed significantly increased expression levels of IL-1β, and IL-6 in tumors from mice treated with LTD_4_ or PGE_2_ compared to vehicle treated mice (Fig. 5, panel a). Furthermore, mice treated with LTD_4_ showed increased IL-10 expression in their tumors compared to tumors from control mice. This increase in IL-10 tumor expression was not noticed in tumors from mice treated with PGE_2_. Furthermore, we analyzed different cytokine levels from murine plasma. In good agreement with our data on mRNA cytokine levels, we observed significantly increased in the plasma concentrations of IL-1β and IL-6 in both LTD_4_ and PGE_2_ treated mice compared with the controls and for IL-10 in LTD_4_ treated mice (Fig. 5, panel b). TNF-α levels were significantly increased in PGE_2_-treated mice, whereas only a modest increase was noticed in LTD_4_-treated mice compared with the vehicle-treated group. In addition, LTD_4_ treatment significantly augmented the CXCL1/KC/GRO plasma levels compared with vehicle treatment, while PGE_2_ treatment did not show any effect on the CXCL1/KC/GRO levels. Interestingly, both LTD_4_- and PGE_2_-treated mice showed a decreased level of IFN-γ compared with the control (Fig. 5, panel b). We also examined whether the plasma levels of eicosanoids were altered in LTD_4_- and PGE_2_-treated mice. Concordantly, the plasma levels of CysLTs and PGE_2_ were found to be significantly increased in both LTD_4_- and PGE_2_-treated mice compared with the control vehicle group (Fig. 5, panel c).

### Both LTD_4_ and PGE_2_ treatment increase plasma CysLTs and PGE_2_ secretion

**Fig. 6:**
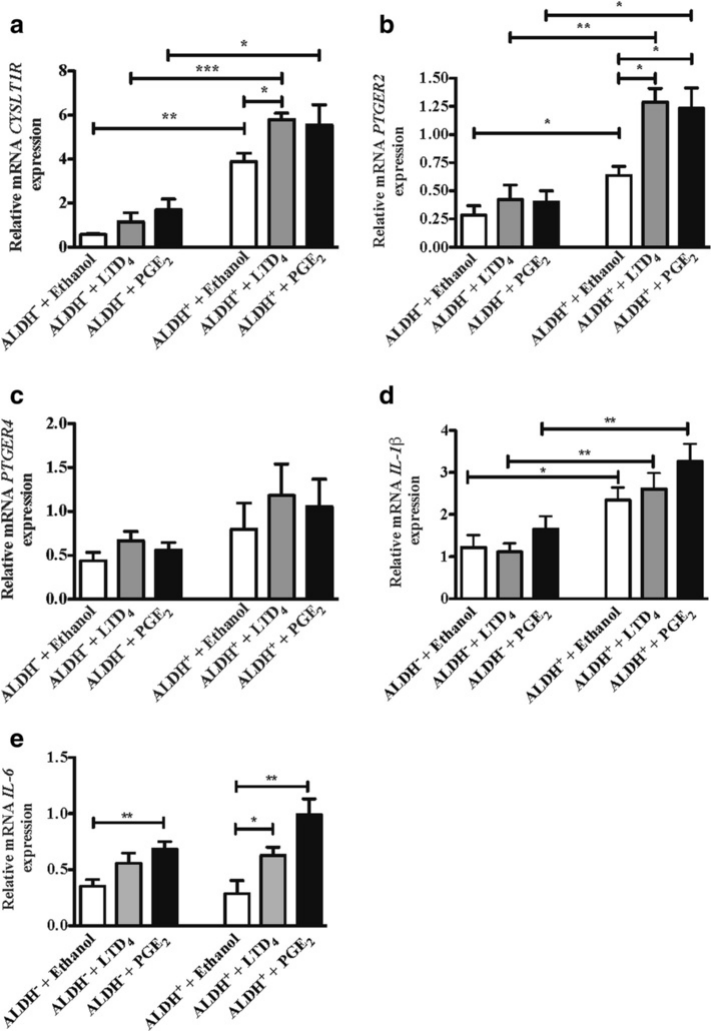
Effect of LTD_4_ or PGE_2_ on CysLTR1, EP2, EP4, IL-1β, and IL-6 in ALDH^−^ and ALDH^+^ HCT-116 cells. **a**–**c** mRNA receptor expression of (**a**) *CYSLT1R*, (**b**) *PTGER2* (EP2), (**c**) *PTGER4* (EP4), (**d**) *IL-1β* and (**e**) *IL-6* mRNA expression in ALDH^−^ and ALDH^+^- HCT-116 cells with vehicle (ethanol), 10 μM of LTD_4_ or PGE_2_ treatment. The results are expressed as means ± SEM, n = 6 mice in each groups. *P < 0.05

As we observed that both LTD_4_ and PGE_2_ drastically induced inflammatory cell infiltration as well as COX-2 up-regulation, we next investigated if LTD_4_ and PGE_2_ triggered an increased expression of their respective receptors, CysLTR1 (*CYSLT1R*), EP2 (*PTGER2*) and EP4 (*PTGER4*). FACS sorted ALDH^+^ and ALDH^−^ HCT-116 were incubated with LTD_4_ or PGE_2_ for 48 h after which we examined the mRNA levels of their respective receptors. CysLTR1 expression was found to be increased in ALDH^+^ cells compared to ALDH^−^ cells. Treatment with LTD_4_ or PGE_2_ increased CysLTR1 expression in both ALDH^+^ and ALDH^−^HCT-116 cells although the levels were significantly higher in ALDH^+^ HCT-116 cells (Fig. 6, panel a). A similar difference in EP2 receptor expression was noticed between ALDH^+^ and ALDH^−^ cells, but the effects of PGE_2_ or LTD_4_ treatment was much more pronounced in ALDH^+^ cells (Fig. 6, panel b). EP4 receptor expression also showed a similar trend of increase as the EP2 receptor expression in ALDH^+^ cells compared to ALDH^−^ cells, however PGE_2_ or LTD_4_ treatment had a trend but no clear effect on EP2 receptor expression in neither ALDH^+^ or ALDH^−^ cells (Fig. 6, panel c). We also examined the effect on *IL-1β* and *IL-6* mRNA levels in these settings (Fig. 6, panel d and e). Interestingly, we found a statistical significant increase in *IL-1β* mRNA levels in ALDH^+^ cells compared to ALDH^−^ cells (Fig. 6, panel d), which indicated the importance of IL-1β in CIC. Furthermore, we found a more pronounced effect of LTD_4_ and PGE_2_ stimulation in ALDH^+^ cells compared to ALDH^−^ cells of the *IL-6* mRNA levels, however no statistical difference between ALDH^+^ and ALDH^−^ cells was seen (Fig. 6, panel e).

## Discussion

CICs, which represent a small population of cancer cells with tumor initiating/stem-like properties, are widely recognized as a significant contributor of CRC development and progression [32]. However, the mechanisms whereby CICs promote tumor growth are largely unknown. Nonetheless, it is presumed that CICs functions are predominantly impacted by their surrounding microenvironment enriched with several mediators, including inflammatory lipid molecules. The present study investigated this possibility to develop a better understanding of the role of the inflammatory lipid mediators LTD_4_ and PGE_2_ in CIC tumor growth [29].

In this study, we demonstrated that the inflammatory mediators LTD_4_ and PGE_2_ augmented tumor growth in mice injected with ALDH^+^ HCT-116 cells, CICs, a finding that is consistent with that in our previous study [29]. Our results show that high ALDH-expressing colon cancer cells show all characteristics of CICs under both *in vivo* and *in vitro* conditions. Additionally, these cells were shown to have increased cancer stem cell characteristics in the presence of the inflammatory mediators LTD_4_ and PGE_2_ [29]. Consistent with this finding, we also observed that the tumor-forming ability of ALDH-sorted CICs was increased markedly with LTD_4_ or PGE_2_ treatment in nude mice. In addition, we noted that ALDH^+^ HCT-116-sorted CICs have higher tumor-initiating ability than parental cells because the onset of tumors was quicker in mice injected with ALDH^+^ HCT-116 sorted cells than in mice injected with parental cells. LTD_4_ and PGE_2_ are known to mediate their responses on colon cancer cells through activation of their receptors, CysLTR1 and EP1-4 [33–35]. Interestingly, we have found that CICs (ALDH^+^) expressed high levels of CysLT1R, EP2 and EP4 receptors and the expression levels of CysLT1 and EP2 receptors were further increased in the presence of LTD_4_ and PGE_2_. These data are in good agreement with our previous findings that the abilities of LTD_4_ or PGE_2_ to promote CIC-induced colony growth is suppressed in the presence of their receptor antagonist [29].

Another in vivo property of CICs is their self-expansion in tumors, and we investigated whether LTD_4_ or PGE_2_ drives the self-expansion of ALDH-sorted cells in tumors. Interestingly, we observed that ALDH proteins were highly expressed in tumors from mice receiving either LTD_4_ or PGE_2_, further substantiating our previous notions that both of these inflammatory mediators play an important role in driving CIC expansion [29]. It was also noteworthy here that the stimulatory effect of the LTD_4_ or PGE_2_ on CIC content was not restricted merely to ALDH-marked cells but to stimulation of the expansion of different CIC markers with similar potency. We crosschecked the data by detecting changes in Dclk1 protein, which is an exclusive marker for cancer stem cells of the intestine. The tumorigenic properties of Dclk1-marked intestinal cancer stem cells have been proven in Apc^Min/+^, suggesting their role in CRC [31].

To identify the potential factors that could be involved in tumor growth induced by LTD_4_ or PGE_2_, we examined some key proteins such as β-catenin, COX-2 and 5-LOX, which are strongly associated with colon cancer cell proliferation and CRC progression [16, 29]. LTD_4_ was demonstrated to trigger the nuclear accumulation of β-catenin in colon cancer cells in vitro, a finding that is linked to the simultaneous up-regulation of COX-2 [16] and the effect of LTD_4_ on proliferation [16]. With this in mind, we examined whether LTD_4_ or PGE_2_ could modify β-catenin signaling in CIC-induced tumors in vivo. Interestingly, we observed a high percentage of β-catenin accumulated in the cytoplasm and nuclear fractions of tumor cells of mice treated with LTD_4_ and PGE_2_. Increased levels of active β-catenin lead to its translocation from the cytosol to the nucleus where it can activate the TCF/LEF family of transcription factors [36]. These transcription factors, in turn, regulate several genes associated with carcinogenesis, such as *cyclin D1*, *c-myc*, and *COX-2* [16]. Interestingly, we detected significantly high COX-2 protein expression within the tumors of mice treated with LTD_4_ or PGE_2_. Elevated COX-2 levels have been demonstrated in all constituent cells of neoplastic colon tissue compared with normal colon tissues [37]. COX-2 overexpression confers resistance to apoptosis and facilitates cell proliferation, which can, together, aggravate a cell’s tumorigenic potential [38–40].

Taken together, our data suggest that inflammatory mediators encourage CICs to evoke tumor growth, possibly by stimulating β-catenin signaling, and concurrent up-regulation of COX-2 as well as proliferation.

Lipid inflammatory mediators, particularly leukotrienes, also play crucial role in leukocyte chemoattraction by the induction of rapid invasion and recruitment of inflammatory cells to the plasma membrane of endothelial cells [10]. Thus, inflammatory cells might be partly involved in driving tumor growth in mice treated with LTD_4_ or PGE_2_. To explore this possibility, we examined the CD45^+^ cell count in tumors of mice receiving LTD_4_ or PGE_2_ treatment. It was noted that both LTD_4_ and PGE_2_ treatment intensified the percentage of CD45^+^ cells in the tumors, with a concurrent increase in ALDH^+^ cells. Furthermore, we found high levels of F4/80-positive staining, representing the total macrophage population within the tumor. In the tumor microenvironment, macrophages play an important role being highly enriched within the tumor as well as in the tumor stroma and secreting many factors known to induce neoplasia [41]. Tumor-associated macrophages (TAMs) play an important role in cancer progression; accordingly, high levels of macrophage infiltration into the tumor tissues are associated with a poor prognosis in cancer patients [42, 43]. These macrophages play a crucial role in tumor immunity and possess potent immunosuppressive functions that contribute to tumor growth. Macrophages were also demonstrated to produce a wide array of cytokines, prostaglandins and leukotrienes [43, 44]. We also found that LTD_4_- and PGE_2_-treated mice had higher plasma levels of CysLTs and PGE_2_, possibly derived from tumor associated macrophages.

In addition, we found significantly higher plasma levels of proinflammatory cytokines IL-1β, IL-2, IL-6 in both LTD_4_- and PGE_2_-treated mice. A significant increase in TNF-α levels was also detected with PGE_2_ treatment. TNF-α, IL-1, and IL-6 have been demonstrated to promote colorectal and colitis-associated tumor development [45]. Interestingly, TNF-α is known to induce β-catenin nuclear accumulation without APC mutations in gastric tumors [46]. Thus, an increased cytokine level could be a possible explanation for driving the increased nuclear accumulation of β-catenin observed in tumors of mice treated with LTD_4_ or PGE_2_. Furthermore, increased levels of the chemokine CXCL1/KC/GRO were found in mice treated with LTD_4_. CXCL1/KC/GRO was shown to recruit and activate murine neutrophils and could modify tumor growth by numerous mechanisms [47]. One interesting finding here is that mice receiving LTD_4_ or PGE_2_ displayed an increased plasma level of IL-10 and a decreased level of IFN-γ. This finding is interesting, particularly because IL-10 is known to inhibit IFN-γ, which has an anti-tumor effect [48]. High production of IL-10 is also a characteristic feature of tumor-associated/M2 macrophages, adapted to suppress immune responses against tumors [43, 49]. These data fits well with our present finding that LTD_4_ as well as PGE_2_ greatly enhanced tumor infiltration by M2 macrophages (recognized by CD206 positive staining) and by increased tumor expression of arginase1. Interestingly, the tumor infiltration of M2 macrophages coincide with an increased tumor expression of the IL-10 in tumors from animals treated with either of the two lipid mediators. In fact, our data support a notion whereby the infiltrating macrophages within the tumor might have acquired a phenotype characterized by a high production of IL-10, which suppresses the anti-tumor actions of IFN-γ and encourages tumor growth. Overall, our data strongly support a tight correlation between host systemic immune responses and tumor growth.

## Conclusions

We have shown that the lipid inflammatory mediators LTD_4_ and PGE_2_ not only stimulated CICs to self-expand but also induced several changes in the CIC tumor microenvironment, which cumulatively drives tumor growth from CICs. This finding deserves considerable attention, and future research should focus not only to target CICs but also associated inflammatory lipid mediators to design more effective therapies for colon cancer prevention.

## Abbreviations

ALDH: Aldehyde dehydrogenase; CICs: cancer initiating cells; COX-2: Cyclooxygenase 2; CRC: Colorectal cancer; IHC: Immunohistochemistry; LTD_4_: Leukotriene D_4_; PGE_2_: Prostaglandin E_2_

## Additional file

Additional file 1: Fig. S1. Immunohistochemistry of LTD_4_- or PGE_2_-treated xenograft mice tumors to demonstrate the 5-LOX protein expression levels. (A) Representative image (40×) of 5-LOX and (B) its corresponding bar graph show overall 5-LOX scores within tumor. The final score represent the sum of the staining intensity and staining percentage within the tumor area. The analyzed data are expressed as mean ± SEM, n = 6 mice in each group. (TIFF 2472 kb)
