# Supplementary figures and images for: The eicosanoids leukotriene D4 and prostaglandin E2 promote the tumorigenicity of colon cancer-initiating cells in a xenograft mouse model
Source: BMC Cancer. 2016 Jul 7;16:425. doi: 10.1186/s12885-016-2466-z (PMC4937611; doi:10.1186/s12885-016-2466-z)

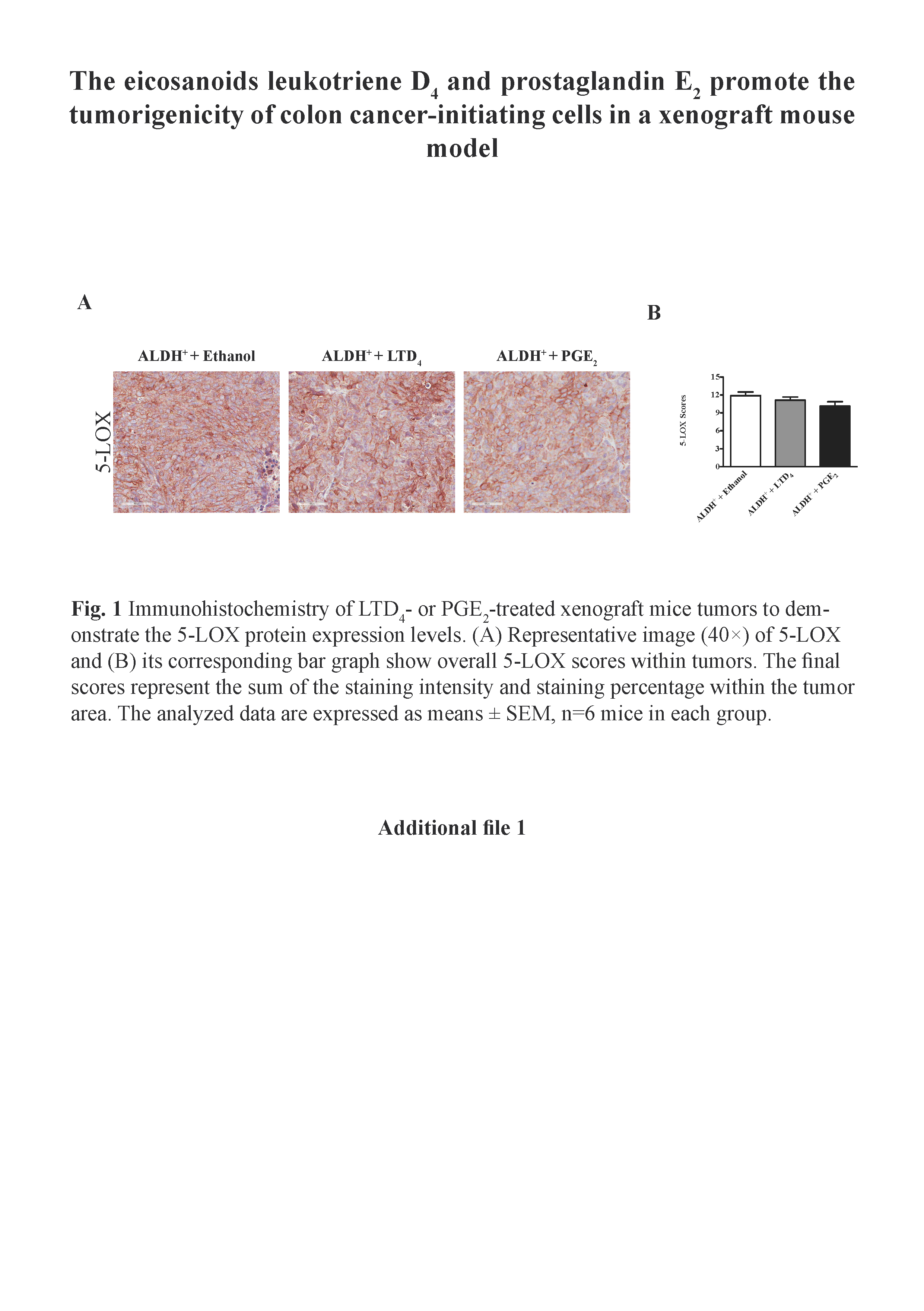

Supplement: Additional file 1: Fig. S1. — Immunohistochemistry of LTD4- or PGE2-treated xenograft mice tumors to demonstrate the 5-LOX protein expression levels. (A) Representative image (40×) of 5-LOX and (B) its corresponding bar graph show overall 5-LOX scores within tumor. The final score represent the sum of the staining intensity and staining percentage within the tumor area. The analyzed data are expressed as mean ± SEM, n = 6 mice in each group. (TIFF 2472 kb) [file 12885_2016_2466_MOESM1_ESM.tiff]
